# Single-Center Experience with the Balloon-Expandable Myval Transcatheter Aortic Valve System in Patients with Bicuspid Anatomy: Procedural and 30-Day Follow-Up

**DOI:** 10.3390/jcm13020513

**Published:** 2024-01-17

**Authors:** Balázs Magyari, Bálint Kittka, Ilona Goják, Kristóf Schönfeld, László Botond Szapáry, Mihály Simon, Rudolf Kiss, Andrea Bertalan, Edit Várady, András Gyimesi, István Szokodi, Iván Gábor Horváth

**Affiliations:** 1 Heart Institute, Medical School, University of Pécs, 13 Ifjuság Str., H-7624 Pécs, Hungary; kittka.balint@pte.hu (B.K.); gojak.ilona@pte.hu (I.G.); schonfeld.kristof@pte.hu (K.S.); szapary.laszlo.botond@pte.hu (L.B.S.); simon.mihaly@pte.hu (M.S.); kiss.rudolf@pte.hu (R.K.); bertalan.andrea@pte.hu (A.B.); istvan.szokodi@pte.hu (I.S.); ivan.g.horvath@aok.pte.hu (I.G.H.); 2 Szentágothai Research Centre, University of Pécs, H-7624 Pécs, Hungary; 3 Department of Medical Imaging, Medical School, University of Pécs, H-7624 Pécs, Hungary; varady.edit@pte.hu; 4 EconNet Research Group, Faculty of Business and Economics, University of Pécs, H-7624 Pécs, Hungary; gyimesi.andras@ktk.pte.hu

**Keywords:** TAVI, bicuspid aortic valve, balloon expendable transcatheter heart valve, paravalvular leak, annular rupture, permanent pacemaker implantation

## Abstract

**Aims:** To report our single-center data regarding the initial 52 consecutive patients with a bicuspid aortic valve who underwent a Transcatheter Aortic Valve Implantation (TAVI) procedure using the new balloon-expandable MYVAL system. The focus is on reporting procedural details and outcomes over the 30-day postoperative period. **Methods:** From December 2019 to July 2023, 52 consecutive patients underwent a TAVI procedure with bicuspid anatomy. All patients had moderate to-high surgical risk or were unsuitable for surgical aortic valve replacement based on the Heart Team’s decision. Outcomes were analyzed according to the VARC-2 criteria. The results of bicuspid patients were compared to patients with tricuspid anatomy in the overall study group, and further analysis involved a comparison between 52 pairs after propensity score matching. The device performance was evaluated using transthoracic echocardiography. Data collection was allowed by the Local Ethical Committee. **Results:** The mean age was 71 ± 7.1 years, and 65.4% were male. The mean Euroscore II and STS score were 3.3 ± 3.2 and 5.2 ± 3.3, respectively. Baseline characteristics and echocardiographic parameters were well balanced even in the unmatched comparison. Procedures were significantly longer in the bicuspid group and resulted in a significantly higher ARI index. All relevant anatomic dimensions based on the CT scans were significantly higher in bicuspid anatomy, including a higher implantation angulation, a higher rate of horizontal aorta and a higher proportion of patients with aortopathy. In the unmatched bicuspid vs. tricuspid comparison, postprocedural outcomes were as follows: in-hospital mortality 0% vs. 1.4% (*p* = 0.394), device success 100% vs. 99.1% (*p* = 0.487), TIA 1.9% vs. 0% (*p* = 0.041), stroke 1.9% vs. 0.9% (*p* = 0.537), major vascular complication 3.8% vs. 2.3% (*p* = 0.530), permanent pacemaker implantation 34% vs. 30.4% (*p* = 0.429), and cardiac tamponade 0% vs. 0.5% (*p* = 0.624). In the propensity-matched bicuspid vs. tricuspid comparison, postprocedural outcomes were as follows: in-hospital mortality 0% vs. 0%, device success 100% vs. 100%, TIA 1.9% vs. 0% (*p* = 0.315), stroke 1.9% vs. 0.9% (*p* = 0.315), major vascular complication 3.8% vs. 0% (*p* = 0.475), permanent pacemaker implantation 34% vs. 24% (*p* = 0.274), and cardiac tamponade 0% vs. 0%. There was no annular rupture nor need for second valve or severe aortic regurgitation in both the unmatched and matched comparison. The peak and mean aortic gradients did not differ at discharge and at 30-day follow-up between the two groups regardless of whether the comparison was unmatched or matched. There were no paravalvular leakages (moderate or above) in the bicuspid patients. Intermediate and extra sizes of the Myval THV system used a significantly higher proportion in bicuspid anatomy with a significantly higher oversize percentage in tricuspid anatomy. **Conclusions:** The TAVI procedure using the Myval THV system in patients with significant aortic stenosis and bicuspid aortic valve anatomy is safe and effective. Hemodynamic parameters do not differ between tricuspid and bicuspid patients. However, the permanent pacemaker implantation rate is higher than expected; its relevance on long-term survival is controversial.

## 1. Introduction

The bicuspid aortic valve (BAV) is the most common congenital defect with a prevalence ranging between 0.5 and 2.0% [1] and male predominancy (3:1). Classified into three types, according to Sievers [2], the BAV shows geographical variability, with type 1 dominance in Europe/USA and type 0 in China [3]. BAV anatomy frequently coexists with enlarged aortic dimensions [4], and due to the asymmetricity-induced increased wall shear stress confirmed by MRI [5], aortic stenosis or aortopathy may develop at a younger age.

Excluded from pivotal TAVI trials, comparisons with surgical aortic valve replacement (SAVR) rely on mainly registries and observational studies. Regarding this, although comparable results could be detected, the BAV has only a class 2b guideline recommendation for TAVI [6]. Selection between balloon-expandable or self-expandable devices presents a challenge in BAV patients. Balloon-expandable valve (BEV) devices with higher radial force may result in more circular expansion, thereby reducing the incidence and severity of paravalvular leak (PVL) but have a higher rate of annular rupture [7]. Self-expandable valve (SEV) devices may be associated with a higher PVL rate and a lower success rate (valve embolism, need for second valve) but have a lower annular rupture rate [8].

It should be mentioned that early- and new-generation SEV and BEV devices impact safety endpoints in favor of new-generation devices [3]. BAV patients are mainly in the low surgical risk profile due to their younger age. Therefore, long-term valve durability of SEV or BEV systems should be confirmed to be an acceptable alternative to SAVR. The asymmetrical shape of the aortic annulus and localization of calcification may result in an inappropriate transcatheter heart valve (THV) expansion and stent distortion. These disturbances elevate valvular shear stress, which negatively impacts valve durability. Consequently, impaired THV leaflet motions may lead to induced hypoattenuating leaflet thickening (HALT) or hypoattenuation affecting motion (HAM) [9,10,11]. Moreover, patient-prosthesis mismatch can occur more frequently when severe malapposition exists [12]. According to Qiu et al., the eccentricity of the THV and the stent distortion are more specific with self-expandable THV devices when implanted in BAV anatomy [13].

This study reports Valve Academic Research Consortium-2 definitions (VARC-2) for our early postprocedural and 30-day experience with the Myval THV system (Meril Life Sciences Pvt. Ltd., Vapi, India) in patients with BAV. Additionally, we conducted an analysis to assess the potential advantages of the intermediate valve sizes of this novel balloon-expandable THV device, particularly in terms of achieving optimal prosthesis sizing.

## 2. Methods

### 2.1. Study Design

This study is a single-center experience. Data were collected retrospectively but recorded systematically in our centralized electronic medical data collecting system (e-MedSolution system) as an integral component of routine care; therefore, this study can be considered real-time, online data collection. Data collection was approved by the Local Ethical Committee (9435-PTE 2022).

### 2.2. Patient Population

We report all Myval cases performed from November 2019 to July 2023 in patients with significant aortic stenosis and BAV anatomy. During the examined period, we used other THV devices (Portico-Abbott, CoreValve-Medtronic, Acurate-Boston Scientific); however, when BAV anatomy was verified on the computed tomography (CT) scan, the Myval THV system was used as the default TAVI device for BAV disease. Notably, the COVID-19 pandemic did not adversely impact patient screening and the execution of TAVI procedures during the examined period.

The main exclusion criteria were general criteria for TAVI (not Myval THV-specific). These were acute myocardial infarction within 14 days, left ventricular ejection fraction ≤ 20%, ongoing infection (including COVID-19), hemodynamic instability, contraindication for antiplatelet or anticoagulant therapy, or life expectancy less than 12 months. Ultimately, 52 consecutive patients were enrolled in the study.

Propensity score methodology was employed to mitigate differences in baseline and procedural characteristics between BAV and TAV patients [14]. BAV or TAV were the dependent variables. The propensity-score matching was performed based on the following covariates:

- Age, sex, body mass index;
- Hypertension, diabetes mellitus, dyslipidemia, ischemic heart disease, prior myocardial infarction, prior percutaneous coronary intervention, prior coronary artery bypass grafting, peripheral artery disease, cerebrovascular disease, COPD, permanent pacemaker implantation (PPI), atrial fibrillation;
- Serum creatinin level, serum hemoglobin level, estimated glomerular filtration ratio, mean aortic valve gradient, global left ventricular ejection fraction;
- New York Heart Association (NYHA) functional class, Society of Thoracic Surgery (STS) score, and Euroscore II.

For each cohort, a propensity score for being in the BAV group was calculated using a logistic regression model. The matching was performed using a greedy matching algorithm with a specified caliper distance of 0.20.

High-gradient severe aortic stenosis was the most common diagnosis. In patients with low-gradient aortic stenosis (low-flow, low-gradient, or paradoxical low-flow, low-gradient), the assessment of severity and indication relied upon dobutamine stress echocardiography and/or native aortic valve CT calcium score. All of the patients had NYHA class II or higher and were unsuitable (or had a moderate-to-high risk) for SAVR based on the decision of the Heart Team. Operative risk was calculated using the logistic EuroSCORE II and STS score. Comprehensive details regarding the baseline clinical and echocardiographic characteristics of the study population are shown in Table 1 and Table 2.

### 2.3. Device Description and Procedure

The technical features of Myval THV (Meril Life Sciences Pvt. Ltd., Vapi, India) were described in the Myval-1 study [15]. Briefly, it uses a bovine pericardium leaflet on a nickel–cobalt frame with anticalcification treatment, a unique cell design resulting in higher radial force, and internal and external sealing tissue minimizing PVL. According to the company specification, in addition to the standard sizes (20 mm, 23 mm, 26 mm, 29 mm), intermediate (21.5 mm, 24.5 mm, 27.5 mm) and extra-large valve sizes (30.5 mm, 32 mm) are available; all of these sizes are compatible with the 14 Fr Python sheath. The Navigator balloon catheter system allows active flexion of the distal part with full retrievability of the undeployed THV system and a dog bone-like expansion of the balloon part to stabilize the valve during deployment. We followed the Myval-1 study’s strong recommendation for predilatation of the native aortic valve [15].

All TAVI procedures were performed in a dedicated hybrid operating room and under conscious sedation. We used the transfemoral-first approach policy as a default strategy when feasible. When transfemoral access was unfeasible based on CT scans, femoral lithotripsy therapy was performed before TAVI to prepare the access site for the implantation; therefore, no alternative access site was used. In our center, the patient screening process included coronary angiography before the TAVI procedure. Consequently, if required, percutaneous coronary intervention was carried out in a separate admission prior to TAVI. This approach ensured that no additional procedures impacting the THV implantation were performed during the TAVI procedure. Adjunct pharmacologic therapy included intraoperative activated clotting time-guided heparin treatment followed by antiplatelet monotherapy (clopidogrel 75 mg/day). If anticoagulant therapy was needed, direct oral anticoagulant therapy was our standard choice, which was augmented with clopidogrel only when previous percutaneous coronary intervention coexisted.

Prior to this study, our center had no experience with balloon-expandable THV implantation; only self-expandable devices were used. Therefore, our results were influenced by the learning curve with this technique. It is noteworthy that throughout the study period, the composition of the TAVI team and their respective roles remained constant. Thus, any potential impact on procedural outcomes due to changes in team dynamics during the study period can be ruled out.

### 2.4. Sizing Method

The accurate sizing of the THV devices is a crucial part of the TAVI procedure, especially in BAV anatomy. The interference between the THV devices and the native aortic valve occurs mainly at the level of leaflets [16], and THVs anchor at the raphe(s) level, as highlighted by Iannopollo [17]. As experience in performing TAVI in this patient subgroup continues to expand, enhanced procedural success has been observed particularly with newer-generation devices. Given the anatomical peculiarities associated with BAV, various sizing techniques and algorithms have been developed, relying on information derived from CT scans. These include annular sizing [18], supra-annular sizing [19], balloon sizing [20], the CASPER algorithm [21], and the Level of Implantation at the Raphe (LIRA) method [22]. Each method has its own set of advantages and limitations, yet a definitive expert consensus on the optimal approach remains elusive.

Irrespective of the chosen sizing method, a certain degree of oversizing with a balloon-expandable THV device is mandatory to decrease the patients–prosthesis mismatch and PVL rates. The optimal target for oversizing ranges from 5 to 15 percent, considering that the lower value may result in more pronounced paravalvular leakage, and a higher oversize percentage may elevate the rate of PPI and annular rupture. We aimed to balance these two scenarios (PVL and annular rupture) using the wide sizing scale of this BEV system. The choice of THV size was determined through the annular sizing method, taking into account the extent and location of calcification, which played a significant role in the final decision-making process.

### 2.5. Study Endpoints and Follow-Up

Safety and efficacy parameters were systematically collected before discharge and at 30-day follow-up. As the primary endpoints, safety was evaluated based on periprocedural outcomes, and short-term hemodynamic performance was assessed via transthoracic echocardiography conducted by independent sonographers. As secondary endpoints, the 30-day combined safety endpoints were defined by the Valve Academic Research Consortium-2 (VARC-2). All relevant endpoints were defined according to the VARC-2 definitions [23]. The severity of perioperative aortic regurgitation was evaluated by intraoperative echocardiography, angiography, and measurement of the aortic regurgitation index (ARI), as described previously [24]. Furthermore, to justify the non-standard sizes, patients received intermediate sizes based on the CT scans, and THV sizing was re-evaluated statistically as if only the standard (20, 23, 26, or 29 mm) devices existed. Comprehensive analyses of all relevant clinical outcomes were conducted on both the propensity-score matched and unmatched populations.

## 3. Statistical Methods

GraphPad Prism (version 9.0, GraphPad Software Inc., San Diego, CA, USA) and SPSS Statistics (version 28.0, IBM, Armonk, NY, USA) were used for statistical analysis. All relevant clinical outcomes were analyzed on the propensity score-unmatched and matched groups. Continuous variables are presented as mean ± standard deviation. Baseline characteristics and echocardiographic measurements were compared using a two-sample Student’s *t*-test. Continuous variables associated with procedural outcomes are presented as median (interquartile range) and were compared using the Wilcoxon rank-sum test. Categorical variables were compared using Fisher’s exact test.

## 4. Results

### 4.1. Baseline Characteristics

Details are shown in Table 1. In the examined period, 372 patients underwent the TAVI procedure in our institute. From those, 269 patients were treated using the novel balloon-expandable THV device. Based on the CT scan, tricuspid aortic valve (TAV) anatomy was verified in 217 patients (80.7%) and BAV anatomy was verified in 52 patients (19.3%). After propensity score matching, 52 pairs could be evaluated. During the examined period, there were no patient lost in follow-up.

In the unmatched cohort, patients with BAV were significantly younger (71.0 ± 7.1 vs. 76.0 ± 6.9, *p* < 0.001), had a higher rate of COPD (25.0% vs. 12.4%, *p* = 0.023) and had a lower Euroscore II value (3.3 ± 3.2 vs. 5.2 ± 5.4, *p* = 0.002). Previous coronary artery bypass grafting was significantly higher in the TAV cohort (19.4% vs. 7.7%, *p* = 0.045). The presence of ischemic heart disease and previous percutaneous coronary intervention were numerically higher in the TAV cohort without statistical significance (45.2% vs. 30.7%, *p* = 0.059; 36.4% vs. 23.1%, *p* = 0.068, respectively). However, the calcium score of the aortic valve was deliberately higher in the BAV anatomy without reaching significance (3911 ± 2554 vs. 3238 ± 1682, *p* = 0.081); this difference disappeared after propensity matching (3911 ± 2554 vs. 3574 ± 1769, *p* = 0.444), despite it not being included as a covariate. In the propensity-matched cohort, no significant differences were observed in the examined covariates.

### 4.2. Procedural Outcomes

Procedural parameters are summarized in Table 3 and Figure 3. Transfemoral access was the most frequent (94.2%), with two (3.8%) surgical femoral cut-downs and only a single trans-subclavian implantation (2%). Conscious sedation was the standard of care (92.3%), and general anesthesia was used in the minority (7.7%). No significant differences were observed between BAV and TAV patients concerning mean pre- and post-procedural aortic gradients in either the unmatched or matched comparisons. Procedural parameters did not differ between the BAV and TAV groups except for the longer operation duration in BAV patients (82.6 ± 28.2 min vs. 95.5 ± 34.2 min, *p* = 0.005 for unmatched and 78.5 ± 24.3 min vs. 95.5 ± 34.2 min, *p* = 0.004 for matched), and significantly higher ARI index in the matched BAV group (25.8 ± 8.5 vs. 30.4 ± 9.2, *p* = 0.014). Further insights into the reasons for the prolonged procedure time in the BAV group can be found in our previous work [25].

The BAV cohort exhibited no in-hospital mortality, device failure, THV malapposition, annular rupture, coronary obstruction, or the need for a second THV. No significant differences between the BAV and TAV cohorts occurred in postprocedural outcomes, except one transient ischemic attack (TIA) in the BAV group, leading to a mathematical distinction in the unmatched comparison (1.9% vs. 0.0%, *p* = 0.041). Cerebral embolic protection devices are not funded for the TAVI procedure, so we could not use these systems. Ischemic stroke (non-disabling) was observed in one patient and TIA was observed in another patient; therefore, the stroke and TIA rate was 1.9%. The diagnosis of TIA/stroke was confirmed by a neurologist based on the clinical findings and CT angiography.

In our study population, 25 patients already had a PPI before the TAVI procedure (23 patients in the TAV group and two in BAV). Data from patients who underwent pacemaker implantation before the TAVI procedure were not considered in any analysis related to the cause of permanent pacemaker implantation after TAVI. New PPI was necessary in 17 BAV cases (34%) and 59 TAV cases (30.4%). No significant difference was identified between the two cohorts in the PPI rate for both the unmatched (BAV vs. TAV, 34.0% vs. 30.4%, *p* = 0.429) or matched comparisons (BAV vs. TAV, 34.0% vs. 24.0%, *p* = 0.274). There were significant differences between patients with new PPI in the BAV group (BAV-PPI) versus TAV patients with new PPI (TAV-PPI) in terms of age, Euroscore II, STS score, and the percentage of THV oversizing.

The calcium score of the aortic valve was found to be non-significantly higher in the BAV-PPI group (3975 ± 2310 vs. 3112 ± 1568, *p* = 0.087). However, these differences disappeared after propensity matching. Excluding patients with PPI prior to the TAVI procedure, no significant differences were observed between BAV-PPI and TAV-PPI patients in implantation depths in both unmatched or matched comparisons. The rate of calcium in the left ventricular outflow tract (LVOT) was similar between BAV-PPI and TAV-PPI patients. On the other hand, in the entire Myval cohort (including BAV and TAV patients), the rate of calcium in the LVOT tract was significantly higher in patients with new PPI after the TAVI procedure (47.4% vs. 30.5%, *p* = 0.009).

Beyond the mentioned findings, no additional differences were identified between patients with or without PPI, including implantation depths, regardless of whether the comparison was unmatched or matched. Distribution of the different THV sizes also did not differ between patients with or without PPI (except the unmatched TAV-PM vs. BAV-PM comparison) and disappeared after propensity score matching. After categorizing the implantation depth, the majority of patients were implanted 4–6 mm or 6–8 mm without significant differences (*p* = 0.739) between patients with (77.3%) or without (75.4%) PPI (details in Table 4 and Table 5, and Supplementary Table S1). In 10 patients (three BAV and seven TAV patients), PPI was performed based on the latest electrophysiological guidelines due to the new onset of borderline conduction disturbances [26].

Vascular complications occurred in six BAV cases. Among these, two cases were classified as major due to the volume of red blood cell transfusion required, and four cases were considered minor. In minor vascular complication cases, balloon angioplasty resolved the patency of the vascular access site. For TAV cases, vascular complications were observed in 19 cases. Among these, five cases were classified as major (one case involved direct surgical device extraction of the undeployed system, resulting in the patient’s death, and four cases were due to the volume of red blood cell transfusion), and 14 cases were considered minor. In cases of minor vascular complications, the patency of the donor artery could be maintained with balloon angioplasty in 10 cases, with surgical suture in three cases, and with mandatory brachial embolectomy in one case. No significant differences could be detected regarding minor and major vascular complications between the BAV and TAV groups. Furthermore, apart from red blood cell transfusions used for treating vascular complications, no additional bleeding complications were detected.

### 4.3. Distribution of THV Sizes According to Valve Types

In 12 cases (23.1%), standard sizes (23, 26, 29 mm) were implanted. In 40 cases (76.9%), intermediate/extra sizes (21.5, 24.5, 27.5, 30.5, 32 mm) were chosen. The utilization of intermediate/extra-size THVs was significantly higher in bicuspid aortic valve (BAV) cases compared to standard sizes (76.9% vs. 23.1%, *p* < 0.0001). This ratio was similar in the TAV group both in the unmatched (52.5% vs. 47.51%, *p* = 0.337) and matched (55.8% vs. 44.2%, *p* = 0.239) cohorts.

**Figure 1:**
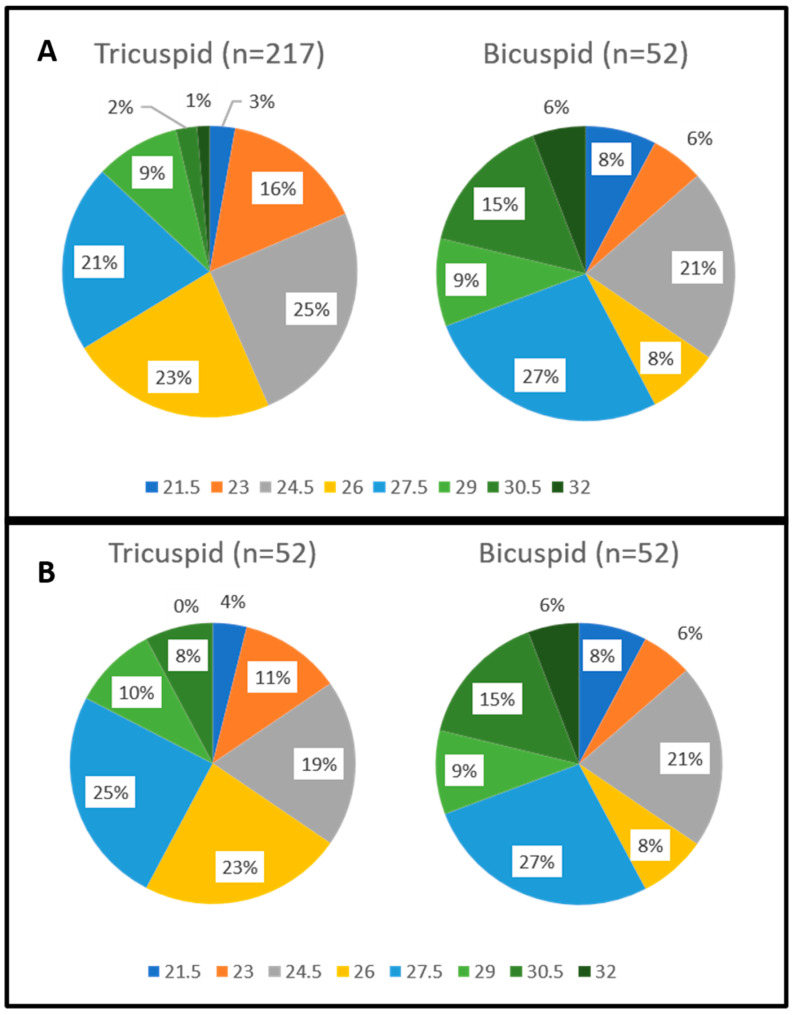
Distribution of the different Myval THV sizes regarding the unmatched comparison (**A**) and after propensity score matching (**B**).

Details are in Table 6 and Table 7 as well as Figure 1. The percentage of oversizing was significantly higher in the TAV patients (6.9 ± 4.4% vs. 4.8 ± 4.7%, *p* = 0.002) compared to BAV patients; this difference persisted after propensity matching (6.4 ± 4.9% vs. 4.8 ± 4.7%, *p* = 0.044). Using the current sizing method, which involves the intention to undersize or nominally size in BAV anatomy, the highest value was 13.8%, and no THV was implanted with oversizing exceeding 15%. On the other hand, when CT scans were re-evaluated as if only standard THV sizes (20, 23, 26, and 29) were available, the percentage of oversizing would have been significantly higher (4.8 ± 4.7% vs. 8.3 ± 9.3%, *p* = 0.017). Moreover, the highest oversizing value would have been 28.6%, and THV would have been implanted with oversizing more than 15% (in 12 cases) or above 20% (in five cases). Additionally, eight patients would have been ineligible for the TAVI procedure using a balloon-expanding THV system due to their anatomical dimensions.

### 4.4. CT Measurements

All relevant details are in Table 8. Patients with BAV anatomy exhibited significantly higher dimensions in all relevant parts of the aortic valve (including LVOT, aortic annulus, sinotubular junction, and ascending aorta) in the unmatched comparison, which was maintained after propensity score matching except for the dimensions of the aortic annulus. However, the eccentricity index of the aortic annulus was identical between the BAV and TAV cohorts. After propensity matching, the LVOT eccentricity was significantly lower in BAV patients than in TAV (0.22 ± 0.06 vs. 0.24 ± 0.05, *p* = 0.043). The implantation angulation and the proportion of horizontal aorta were significantly higher in the BAV cohort. Furthermore, aortopathy (defined as the aortic ascendens above 40 mm in diameter [27]) was markedly more frequent in BAV anatomy (*p* < 0.0001).

The distribution of the different BAV morphologies using the Sievers classification was as follows: Type 0 in 13 (25%) patients, Type 1a in 33 (63.5%) patients, Type 1b in 5 (9.6%) patients, and Type 2 in one (1.9%) patient. Based on CT scans from the 39 raphe-type BAV patients, 9 (23%) patients had no calcified raphe or excessive leaflet calcification, 10 (26%) patients had calcified raphe or excessive leaflet calcification, and 20 (51%) patients had calcified raphe plus excessive leaflet calcification. Taking into consideration these CT-based subcategories, as reported by Sung-Han Yoon et al., our BAV patient cohort was at high risk for long-term all-cause mortality [27].

### 4.5. VARC-2 Outcomes at 30-Day Follow-Up

**Figure 2:**
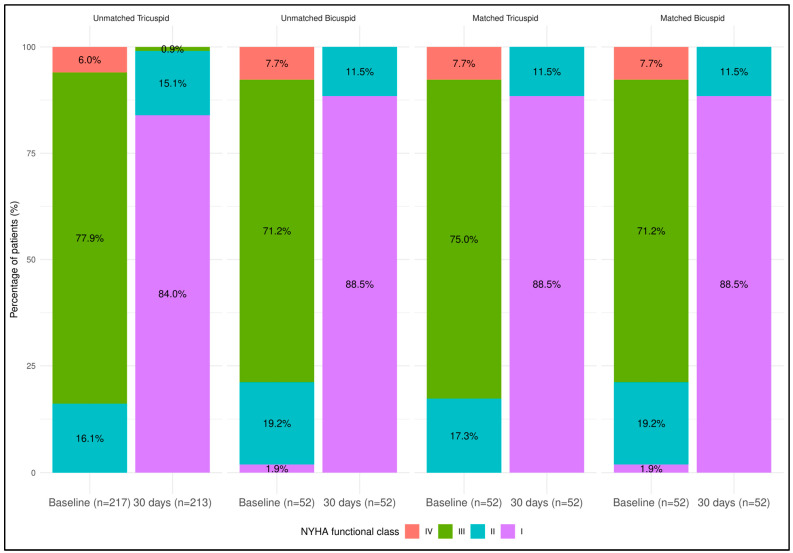
Patient distribution according to NYHA functional class regarding the unmatched tricuspid versus bicuspid comparison and the matched tricuspid versus bicuspid comparison. NYHA functional class improved significantly in every patient cohort from baseline to 30-day follow-up (patient at NYHA III/IV vs. NYHA I/II, *p* < 0.0001 for all comparison).

**Figure 3:**
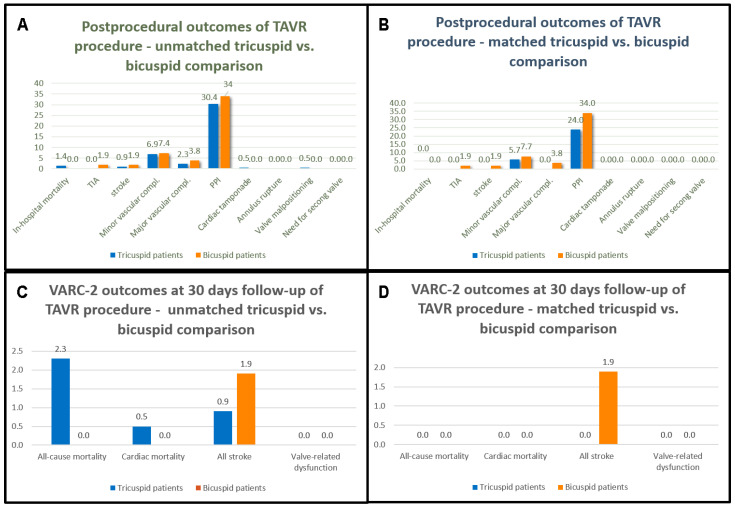
Postprocedural outcomes (<72 h after the index procedure) of the study population regarding the unmatched comparison (**A**) and after propensity score matching (**B**). VARC-2 outcomes at 30-day follow-up regarding the unmatched comparison (**C**) and after propensity score matching (**D**). Values are in percentage.

Details are listed in Table 9 and Table 10 as well as Figure 2 and Figure 3. At 30 days, there were no deaths in the matched comparison. In the unmatched TAV group, there were two cases of death (one case due to COVID-19 pneumonia and one case due to life-threatening bleeding), resulting in 2.3% all-cause mortality and 0.5% cardiac mortality. During the 30-day follow-up period after patient discharge, there were no new strokes, no need for new PPI, and no valve-related dysfunction requiring repeat procedures. Additionally, at 30 days, no bleeding complication, prosthetic valve endocarditis/thrombosis or thrombo-embolic events were detected. A significant improvement in NYHA functional class was observed with no patients at NYHA functional class III in the BAV cohort and only two in the unmatched TAV cohort.

### 4.6. Echocardiographic Outcomes

**Figure 4:**
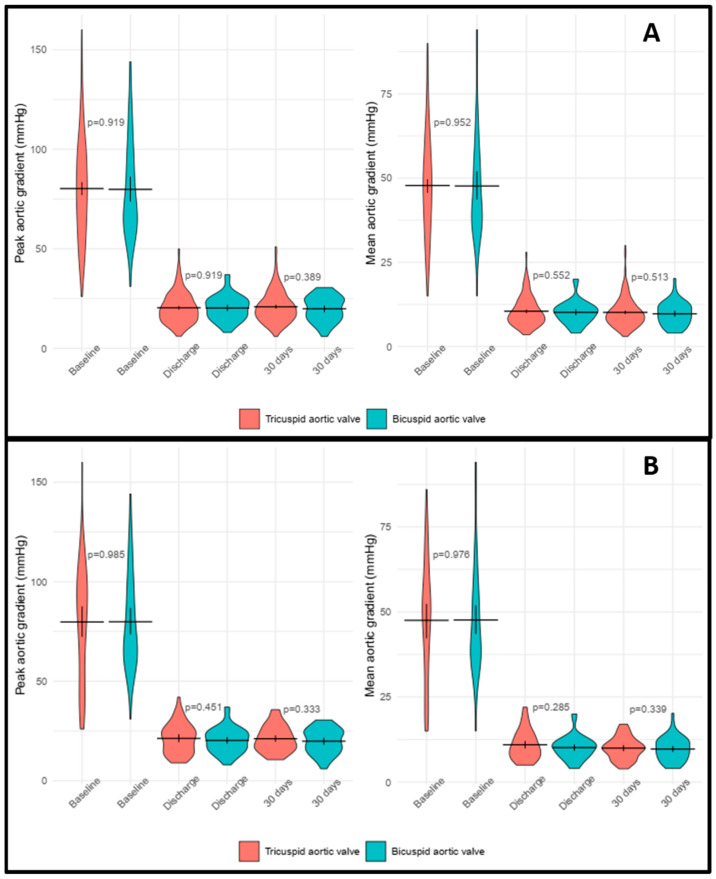
Peak and mean aortic gradients at the different time period regarding the unmatched comparison (**A**) and after propensity score matching (**B**).

Detailed data are in Table 11 and Figure 4. Baseline echocardiographic parameters included mean left ventricle ejection fraction (LVEF), mean aortic peak velocity, peak aortic transvalvular gradient (pAVG), mean aortic transvalvular gradient (mAVG), and the proportion of patients with mitral or tricuspid regurgitation grade III or above. These parameters were identical regardless of unmatched or matched comparison. After the TAVI procedure, no significant difference could be detected between TAV and BAV cohorts in LVEF, pAVG, and mAVG at discharge and 30-day follow-up. Paravalvular leakage (grade moderate or above) was absent in the BAV patients and was present in a minority of TAV patients. These favorable echocardiographic results occurred in both unmatched and matched comparisons.

## 5. Discussion

While there are clear recommendations for performing TAVI in different surgical risk categories, caution should be exercised when extrapolating these results to patients with a bicuspid aortic valve (BAV) due to their more heterogeneous anatomy. The present study was conducted to analyze the safety, efficacy, procedural, and clinical outcomes of the TAVI procedure using the novel balloon-expandable THV device in BAV anatomy and to compare the results to patients with TAV aortic stenosis. The major findings are as follows:

1. The TAVI procedure with the Myval THV system is safe with no significant differences in VARC-2 outcomes in the unmatched and propensity score-matched comparisons.
2. The TAVI procedure with the Myval THV system is effective with no significant differences in hemodynamic performance between BAV and TAV patients based on invasive and non-invasive measurements. Moreover, ARI was significantly higher after the BAV TAVI procedure.
3. The sizing method employed, in conjunction with the broad range of standard and intermediate/extra sizes, facilitated the suitability of all patients for the TAVI procedure irrespective of their anatomical dimensions. Also, there was a significantly lower rate of oversizing in BAV patients compared to TAV.
4. Beyond the absence of major mechanical complications, the rate of PPI was high in both groups. No significant differences in risk factors were detected between BAV and TAV patients with post-TAVI PPI except for a tendency for higher aortic valve calcium scores in BAV patients in the unmatched comparison. Therefore, this high PPI rate seems to be more related to patient characteristics than the device used. The significantly higher proportion of LVOT calcium in the PPI group of the whole patient cohort may underscore this theory.

While these results are comparable to the PARTNER 3 Bicuspid Registry, it should be emphasized that the registry excluded patients with severe LVOT and raphe calcification and patients with an ascending aorta diameter of over 40 mm. The high success rate in this study (with the absence of fatal mechanical complications and the lower postprocedural gradients compared to this registry) highlights our results even in our all-comer, unselected, high-estimated-TAVI-risk patient population. The favorable echocardiographic findings included the lack of a moderate or above PVL grade, which may be attributed to our intentional approach of sizing nominally or undersizing with non-standard THV sizes.

Reported PPI rates after TAVI vary widely (2.3–36.1% [28,29]), but our PPI rate seems high. Considering the “calcification effect,” no differences could be detected between patients with or without pacemaker implantation except a tendency for higher aortic valve calcium score in BAV patients and the significantly higher proportion of calcium in the LVOT in patients with new PPI. Data regarding the impact of the aortic valve calcium score and the existence of calcium in the LVOT on PPI are controversial.

Comparing our PPI results to the patient cohort of Delgado-Arana et al., our patient cohort had a significantly higher Agatston aortic valve calcium score (3374 ± 1174) and a higher rate of calcium presence in the LVOT (47.4% in the PPI group—details above) at identical implantation depths. In their patient cohort, the low incidence of new PPI was associated with a mean calcium score of 2314, and only 8.4% had calcium in the LVOT [30]. Furthermore, distribution differences in the THV sizes between our patients with or without PPI were detected only in the TAV-PM versus BAV-PM comparison (which disappeared after propensity score matching). This difference resulted from a significantly higher proportion of intermediate/extra sizes in the whole BAV cohort compared to the TAV cohort. Considering these data, our high PPI rate seems to be due to patient characteristics rather than device-related.

This study was conducted to analyze safety, efficacy, and postprocedural results. Therefore, this study could not evaluate THV durability, considering the short follow-up period. According to Sung-Han Yoon et al., most differences in outcomes between TAV and BAV patients are present in procedural outcomes and in the short term (30 days), while later differences are not obvious [31]. Based on these findings, we anticipate acceptable long-term results; however, this is hypothetical, and longer follow-up periods are needed.

There were excellent surgical results in aortic stenosis with BAV anatomy. However, patient and device selection will be crucial for the TAVI procedure to serve as a real competitor against surgical techniques. Due to the lower average age in the BAV group, there may be an increased need for re-intervention. When surgical explanation of a TAVI device is needed, mortality is higher than in patients with redo-SAVR due to the need for intensive endarterectomy and/or aortic root repair [32]. Therefore, valve durability is a major issue in this subset of patients. Especially in the valve-in-valve procedure, implanting a supra-annular THV into an intra-annular THV system seems more reasonable than the reverse situation for coronary access. In Makkare et al., all annular ruptures occurred in patients with Type 1 BAV with calcified raphe; therefore, these anatomical properties should be accounted for when implanting a BEV system into BAV anatomy [33].

The BAVARD registry highlighted the importance of THV underexpansion (which may be responsible for valve durability and leaflet thrombosis). It emphasized choosing the appropriate THV size to avoid the failing to achieve the maximal diameter and circularity of the THV device [19]. The wide sizing scale in this study made this novel BEV system a safe and effective alternative THV system.

## 6. Study Limitations

Propensity score matching is a well-accepted approach in observational research to avoid the potential impact of the difference between different cohorts, but bias may occur. There was no independent center to verify adverse events and assess the existence and grade of paravalvular leakage. The short follow-up period may be a limitation, and long-term follow-up is essential.

## 7. Conclusions

The novel balloon-expandable THV system is safe and effective in BAV patients compared to TAV for procedural outcomes and 30-day follow-up. Beyond standard sizes, the intermediate and extra sizes have additional value in BAV anatomy, as evidenced by the lack of annular rupture and the absence of moderate-or-above PVL grade. This technology combines the known positive features of BEV and SEV systems without their negative aspects. Based on our results, the high PPI rate was likely attributable to patient characteristics rather than the device used.

## Figures and Tables

**Table 1:** Baseline demographics and clinical parameters of the whole study population and after propensity score matching. St.p. MI: previous myocardial infarction, St.p. PCI: previous percutaneous coronary intervention, St.p. CABG: previous coronary artery bypass grafting, COPD: chronic obstructive pulmonary disease, BAV: balloon aortic valvuloplasty, St.p. MVR: previous mitral valve replacement, St.p. AVR: previous aortic valve replacement, NA: not added value. *: statistically significant difference.

Baseline Characteristic of Study Population (n = 269)		Tricuspid (n = 217)	Bicuspid (n = 52)	*p* Value	Tricuspid (n = 52)	Bicuspid (n = 52)	*p* Value
**Age (years)**	75 ± 7.3	76 ± 6.9	71 ± 7.1	<0.001	72.3 ± 8.9	71 ± 7.1	0.430
**Men (n/%)**	165 (61.3)	131 (60.4%)	34 (65.4)	0.505	37 (71.1)	34 (65.4)	0.527
**Body mass index (kg/m^2^)**	29 ± 5.3	29.3 ± 5.3	28.1 ± 5.4	0.165	28.1 ± 5.3	28.1 ± 5.4	0.997
**Body surface area (m^2^)**	1.94 ± 0.2	1.95 ± 0.24	1.92 ± 0.23	0.254	1.95 ± 0.26	1.92 ± 0.23	0.487
**Hypertension**	262 (97.4)	211 (97.2)	51 (98.1)	0.732	51 (98.1)	51 (98.1)	1.0
**Diabetes mellitus**	116 (43.1)	98 (45,2)	18 (34,6)	0.168	17 (32.7)	18 (34.6)	0.836
**Hyperlipidemia**	243 (90.3)	197 (90.8)	46 (88.5)	0.611	46 (88.5)	46 (88.5)	1.0
**NYHA class III or IV**	223 (82.9)	182 (83.9)	41 (78.8)	0.387	43 (82.7)	41 (78.8)	0.619
**Ischemic Heart Disease**	114 (42.4)	98 (45.2)	16 (30.7)	0.059	17 (32.7)	16 (30.8)	0.833
**St.p. MI**	62 (23)	53 (24.4)	9 (17.3)	0.274	9 (17.3)	9 (17.3)	1.0
**St.p. PCI**	91 (33.8)	79 (36.4)	12 (23.1)	0.068	13 (25.0)	12 (23.1)	0.819
**St.p. CABG**	46 (17.1)	42 (19.4)	4 (7.7)	* 0.045	4 (7.7)	4 (7.7)	1.0
**Peripheral artery disease**	40 (14.9)	31 (14.3)	9 (17.3)	0.582	8 (15.4)	9 (17.3)	0.791
**Cerebrovascular disease**	38 (14.1)	32 (14.7)	6 (11.5)	0.551	4 (7.7)	6 (11.5)	0.506
COPD	40 (14.9)	27 (12.4)	13 (25.0)	* 0.023	10 (19.2)	13 (25.0)	0.478
**Previous BAV**	10 (3.7)	9 (4.1)	1 (1.9)	0.446	3 (5.8)	1 (1.9)	0.308
**Permanent PM**	25 (9.3)	23 (10.6)	2 (3.8)	0.132	2 (3.8)	2 (3.8)	1.0
**Atrial fibrillation**	53 (19.7)	46 (21,2)	7 (13.5)	0.208	9 (17.3)	7 (13.5)	0.587
**Logistic EuroSCORE (%)**	14.9 ± 14.4	15.5 ± 15.2	12.2 ± 10.4	0.131	11.3 ± 10.8	12.2 ± 10.4	0.663
**Logistic EuroSCORE II (%)**	4.8 ± 5.1	5.2 ± 5.4	3.3 ± 3.2	* 0.002	3.2 ± 2.5	3.3 ± 3.2	0.770
**STS score (%)**	6.2 ± 4.3	6.4 ± 4.4	5.2 ± 3.3	0.069	4.7 ± 3.9	5.2 ± 3.3	0.505
**Calcium score of the aortic valve**	3374 ± 1174	3238 ± 1682	3911 ± 2554	0.081	3574 ± 1769	3911 ± 2554	0.444
**Serum creatinine (umol/L)**	101.8 ± 44.8	103.5 ± 48.1	94.6 ± 25.8	0.199	96.4 ± 31.3	94.6 ± 25.8	0.754
**Estimated GFR (mL/min)**	67.6 ± 26.4	66.6 ± 27.2	71.9 ± 22.9	0.188	72.3 ± 29.2	71.9 ± 22.9	0.952
**St.p. MVR**	4 (1.5)	4 (1.8)	0 (0.0)	0.324	0 (0.0)	0 (0.0)	NA
**St.p. AVR**	1 (0.4)	1 (0.5)	0 (0.0)	0.624	0 (0.0)	0 (0.0)	NA
**Dialysis**	3 (1.1)	2 (0.9)	1 (1.9)	0.537	0 (0.0)	1	0.315
**Procedure status**							
**elective**	253 (94)	204 (94)	49 (94.2)	0.952	48 (92.3)	49 (94.2)	0.696
**urgent**	15 (5.6)	12 (5.5)	3 (5.8)	0.946	3 (5.8)	3 (5.8)	1.0
**acut**	1 (0.4)	1 (0.5)	0 (0.0)	0.624	1 (1.9)	0 (0.0)	0.315

**Table 2:** Echocardiographic parameters of the study population of the whole study population and after propensity score matching.

	Unmatched (n = 269)			Matched (n = 52)		
Baseline Echocardiographic Parameters of the Study Population (n = 269)	Tricuspid (n = 217)	Bicuspid (n = 52)	*p* Value	Tricuspid (n = 52)	Bicuspid (n = 52)	*p* Value
***Mean LVEF***	55.5 ± 12.8	54.2 ± 13.6	0.543	54.8 ± 14.1	54.3 ± 13.6	0.827
***Mean AoVmax* (m/s)**	4.4 ± 0.7	4.4 ± 0.7	0.966	4.4 ± 0.8	4.4 ± 0.7	0.919
***Aortic peak gradient* (Hgmm)**	80.2 ± 25.7	79.9 ± 24.1	0.928	79.7 ± 29.1	79.9 ± 24.1	0.975
***Aortic mean gradient* (Hgmm)**	47.8 ± 15.5	47.6 ± 15.9	0.949	47.6 ± 17.5	47.6 ± 15.8	0.988
***Mitral insufficiency III or IV***	45	7	0.233	7	7	1.0
***Tricuspid insufficiency III or IV***	38	9	0.972	8	9	0.791

**Table 3:** Procedural parameters of the study population of the whole study population and after propensity score matching. ARI: aortic regurgitation index. NA: not added value. *: statistically significant difference.

		Unmatched (n = 269)			Matched (n = 52)		
Variable	*Overall* *(n = 269)*	*Tricuspid* *(n = 217)*	*Bicuspid (n = 52)*	*p Value*	*Tricuspid* *(n = 52)*	*Bicuspid* *(n = 52)*	*p Value*
**Type of anesthesia**							
***general***	15	11	4	0.459	0	4	* 0.041
***local***	255	207	48	0.369	52	48	* 0.041
**Access site**							
***femoral (percutaneous)***	265	216	49	* 0.005	52	49	0.079
***femoral (surgical)***	2	0	2	* 0.004	0	2	0.153
***subclavia***	2	1	1	0.270	0	1	0.315
***axillaris***	0	0	0	NA	0	0	NA
***direct aortic***	0	0	0	NA	0	0	NA
***Contrast agent***	225.3 ± 99.5	224.5 ± 100.2	230 ± 97.4	0.719	218.7 ± 92.1	230 ± 97.4	0.544
***Operation duration (min)***	85.4 ± 29.5	82.6 ± 28.2	95.5 ± 34.2	* 0.005	78.5 ± 24.3	95.5 ± 34.2	* 0.004
***Predilatation***	269	217	52	1.000	52	52	1.000
***Postdilatation***	34	28	6	0.790	6	6	1.000
***Preimpl. mean AV gradient***	53.6 ± 18.2	53.9 ± 18.5	52.6 ± 17.0	0.658	55.4 ± 19.9	52.6 ± 17	0.452
***Postimpl. mean AV gradient***	6.4 ± 6.2	6.4 ± 6.3	6.3 ± 5.8	0.942	6.1 ± 5.5	6.3 ± 5.8	0.868
***ARI***	29 ± 18.7	28.7 ± 20.5	30.4 ± 9.2	0.576	25.8 ± 8.5	30.4 ± 9.2	* 0.014

**Table 4:** Data regarding patient with and without PM implantation regarding the unmatched cohort (all patients received Myval THV in the study period) and matched cohort (52 patients with bicuspid and 52 patients with tricuspid aortic valve anatomy).

	Unmatched (n = 269)			Matched (n = 104)		
	Non PM (n = 193)	PM (n = 76)	*p*-Value	Non PM (n = 75)	PM (n = 29)	*p*-Value
**Age**	75.1 ± 6.9	74.8 ± 8.1	0.712	71.8 ± 7.3	71.1 ± 9.8	0.672
**Euroscore**	15.7 ± 15.5	12.7 ± 11	0.077	12.1 ± 11	10.8 ± 9.3	0.567
**Euroscore II**	5.1 ± 5.4	4.3 ± 4.1	0.286	3.3 ± 2.9	3.2 ± 2.8	0.902
**STS score**	6.1 ± 3.8	6.2 ± 5.2	0.993	4.9 ± 3	5.1 ± 5	0.817
**Ca score**	3393 ± 1943	3304 ± 1778	0.737	3687 ± 2177	3880 ± 2253	0.694
**Ca in LVOT**	59 (30.5%)	36 (47.4%)	*0.009*	26 (34.7%)	13 (44.8%)	*0.337*
**Oversizing**	6.3 ± 4.7	6.8 ± 4.1	0.490	5.5 ± 5	5.8 ± 4.4	0.723
**THV implantation depth**						
***Left coronary side* (mm)**	5.4 ± 2.1	5.7 ± 1.9	0.254	5.4 ± 2.1	5.5 ± 2.1	0.886
***Non coronary side* (mm)**	6.2 ± 1.9	6.3 ± 1.8	0.831	6.2 ± 2.1	6.1 ± 2.2	0.939
***Right coronary side* (mm)**	5.9 ± 1.9	6.1 ± 1.8	0.506	6 ± 1.9	6 ± 2.0	0.884
***Average depth* (mm)**	5.8 ± 1.9	6.0 ± 1.8	0.477	5.9 ± 1.9	5.9 ± 2	0.943
**THV size**						
***21.5***	8	2	0.555	4	2	0.759
***23***	26	11	0.830	7	2	0.692
***24.5***	48	17	0.666	16	5	0.641
***26***	38	15	0.993	13	3	0.376
***27.5***	42	17	0.914	18	9	0.463
***29***	17	8	0.662	8	2	0.559
***30.5***	8	5	0.402	7	4	0.507
***32***	5	1	0.524	2	2	0.314
**Standard size**	81 (41.9%)	34 (44.7%)	*0.680*	28 (37.3%)	7 (24.1%)	0.202
**Intermediate/extra size**	111 (58.1%)	42 (55.3%)	*0.680*	47 (62.7%)	22 (75.9%)	0.202

**Table 5:** Data of comparison between patients with PM implantation and tricuspid aortic valve anatomy (TAV-PM) versus patients with PM implantation and bicuspid aortic valve anatomy (BAV-PM) regarding the unmatched and matched cohort.

	Unmatched (n = 76)			Matched (n = 29)		
	TAV-PM (n = 59)	BAV-PM (n = 17)	*p*-Value	TAV-PM (n = 12)	BAV-PM (n = 17)	*p*-Value
**Age**	75.8 ± 7.8	71.6 ± 8.2	0.031	71.2 ± 12.1	71 ± 8.2	0.977
**Euroscore**	12.9 ± 11.9	11.9 ± 7.4	0.723	9.2 ± 11.7	11.9 ± 7.4	0.451
**Euroscore II**	4.7 ± 4.5	3 ± 2.2	0.042	3.4 ± 3.6	3.0 ± 2.2	0.738
**STS score**	6.7 ± 5.8	4.2 ± 1.1	0.002	6.3 ± 7.7	4.2 ± 1.1	0.356
**Ca score**	3112 ± 1568	3975 ± 2310	0.087	3753 ± 2271	3975 ± 2310	0.802
**Ca in LVOT**	29 (49.1%)	7 (41.2%)	*0.562*	6 (50%)	7 (41.2%)	*0.638*
**Oversizing**	7.3 ± 3.9	4.9 ± 4	0.038	7.1 ± 4.7	4.9 ± 4.0	0.184
**THV implantation depth**						
***Left coronary side* (mm)**	5.6 ± 1.9	5.9 ± 1.9	0.516	4.9 ± 2.4	5.9 v 1.9	0.179
***Non coronary side* (mm)**	6.2 ± 1.9	6.5 ± 1.7	0.515	5.6 ± 2.7	6.5 ± 1.7	0.282
***Right coronary side* (mm)**	6.0 ± 1.9	6.4 ± 1.6	0.411	5.4 ± 2.6	6.4 ± 1.6	0.218
***Average depth* (mm)**	5.9 ± 1.8	6.3 ± 1.6	0.460	5.3 ± 2.5	6.3 ± 1.6	0.205
**THV size**						
***21.5***	1	1	0.342	1	1	0.798
***23***	10	1	0.253	1	1	0.798
***24.5***	14	3	0.596	2	3	0.945
***26***	13	2	0.349	1	2	0.765
***27.5***	12	5	0.429	4	5	0.822
***29***	8	0	0.108	2	0	0.081
***30.5***	1	4	0.001	1	4	0.286
***32***	0	1	0.061	0	1	0.393
**Standard size**	31 (52.5%)	3 (17.6%)	*0.011*	4 (33.3%)	3 (17.6%)	0.331
**Intermediate/extra size**	28 (47.5%)	14 (82.4%)	*0.011*	8 (66.7%)	14 (82.4%)	0.331

**Table 6:** Distribution of different THV sizes regarding the comparison between tricuspid aortic valve and bicuspid aortic valve patients. THV: transcatheter heart valve, standard size: 23, 26, 29; intermediate + extra size: 21.5, 24.5, 27.5, 30.5, 32.

	Unmatched (n = 269)			Matched (n = 52)		
*THV Size*	*Tricuspid (n = 217)*	*Bicuspid (n = 52)*	*p Value*	*Tricuspid (n = 52)*	*Bicuspid (n = 52)*	*p Value*
***21.5***	6	4	0.092	2	4	0.400
***23***	34	3	0.063	6	3	0.295
***24.5***	54	11	0.572	10	11	0.807
***26***	49	4	0.015	12	4	0.030
***27.5***	45	14	0.333	13	14	0.823
***29***	20	5	0.929	5	5	1.0
***30.5***	5	8	<0.0001	4	8	0.220
***32***	3	3	0.054	0	3	0.079
***Oversizing*** **(%)**	6.9 ± 4.4	4.8 ± 4.7	0.002	6.4 ± 4.9	4.8 ± 4.7	0.044
***Standard size***	103 (47.5%)	12 (23.1%)	*0.001*	23 (44.2%)	12 (23.1%)	0.022
***Intermediate/extra size***	113 (52.5%)	40 (76.9%)	*0.001*	29 (55.8%)	40 (76.9%)	0.022
	0.337 *	<0.0001 **		0.239 *	<0.0001 **	

*—level of significance regarding the comparison between tricuspid patient with standard size versus tricuspid patient with intermediate/extra size. **—level of significance regarding the comparison between bicuspid patient with standard size versus bicuspid patient with intermediate/extra size.

**Table 7:** Distribution of different THV sizes in patients with bicuspid aortic valve as they were actually implanted and regarding if they would have implanted after re-evaluation of THV sizes without intermediate sizes. NA: not added value.

THV Size	Bicuspid (n = 52)	Bicuspid Re-Size (n = 52)	*p* Value
***20***	0	1	
***21.5***	4	NA	
***23***	3	9	
***24.5***	11	NA	
***26***	4	16	
***27.5***	14	NA	
***29***	5	18	
***30.5***	8	NA	
***32***	3	8	
***Oversize (%)***	4.76 ± 4.7	8.3 ± 9.3	0.017

**Table 8:** CT parameters of the patients undergoing TAVI with tricuspid and bicuspid anatomy regarding unmatched comparison and after propensity score matching. RCA: right coronary artery, LM: left main, SOV: sinus of valsalva, STJ: sinotubular junction, LVOT: left ventricle outflow tract.

	Unmatched (n = 269)			Matched (n = 52)		
Variable	Tricuspid (n = 217)	Bicuspid (n = 52)	*p* Value	Tricuspid (n = 52)	Bicuspid (n = 52)	*p* Value
***Aortic anulus perimeter***	79.5 ± 6.9	83.9 ± 9.8	**0.003**	81.1 ± 7.1	83.9 ± 9.7	0.097
***Aortic anulus perimeter derived Ø***	25.4 ± 2.2	26.7 ± 3.1	**0.004**	25.8 ± 2.3	26.7 ± 3.1	0.097
***Aortic anulus area***	494 ± 85	551.4 ± 125.4	**0.003**	511.5 ± 89.1	551.3 ± 125.4	0.065
***Aortic anulus area derived Ø***	24.9 ± 2.1	26.3 ± 3.0	**0.004**	25.4 ± 2.2	26.3 ± 3.0	0.090
***Aortic anulus Ø, min***	22.5 ± 2.0	23.6 ± 2.9	**0.013**	22.8 ± 2.2	23.6 ± 2.9	0.155
***Aortic anulus Ø, max***	28.0 ± 2.5	29.3 ± 3.6	**0.025**	28.6 ± 2.6	29.3 ± 3.6	0.270
***Aortic anulus Ø, average***	25.3 ± 2.1	26.4 ± 3.1	**0.013**	25.7 ± 2.3	26.4 ± 3.1	0.186
***Aortic anulus, Eccentricity***	0.20 ± 0.05	0.19 ± 0.07	0.574	0.2 ± 0.05	0.19 ± 0.07	0.490
***RCA height***	16.8 ± 3.1	17.5 ± 3.1	0.194	16.8 ± 2.8	17.5 ± 3.1	0.291
***LM height***	13.5 ± 3	14.9 ± 3.1	0.004	14.5 ± 3.1	14.9 ± 3.1	0.534
***SOV diameter, left***	33.4 ± 3.4	33.3 ± 7.6	0.980	33.9 ± 3.9	33.3 ± 5.3	0.588
***SOV diameter, right***	31.6 ± 3.1	33.4 ± 5.3	**0.040**	31.8 ± 3.3	33.4 ± 5.3	0.097
***SOV diameter, non***	33.1 ± 3.3	36.9 ± 4.9	**<0.0001**	33.3 ± 3.2	36.9 ± 5.0	**<0.0001**
***SOV height***	10.2 ± 1.9	10.7 ± 2.7	0.116	10.2 ± 2	10.7 ± 2.7	0.215
***Ascending aorta diameter, min***	34.0 ± 3.8	39.9 ± 5.5	**<0.0001**	33.9 ± 3.9	39.5 ± 5.5	**<0.0001**
***Ascending aorta diameter, max***	35.7 ± 3.8	41.4 ± 5.8	**<0.0001**	35.6 ± 3.7	41.4 ± 5.8	**<0.0001**
***Ascending aorta diameter, average***	34.8 ± 3.8	40.5 ± 5.6	**<0.0001**	34.7 ± 3.8	40.5 ± 5.6	**<0.0001**
***Ascending aorta perimeter***	109 ± 12.1	125.1 ± 18.3	**<0.0001**	109 ± 12.5	125.1 ± 18.3	**<0.0001**
***Ascending aorta perimeter derived Ø***	34.7 ± 3.9	39.8 ± 5.8	**<0.0001**	34.7 ± 3.9	39.8 ± 5.8	**<0.0001**
***Ascending aorta area***	954.3 ± 215.8	1267.3 ± 362.2	**<0.0001**	954.6 ± 218.1	1267.3 ± 362.2	**<0.0001**
***Ascending aorta area derived Ø***	34.6 ± 3.9	39.7 ± 5.8	**<0.0001**	34.6 ± 3.9	39.8 ± 5.8	**<0.0001**
***STJ Ø, min***	27.9 ± 2.9	32.2 ± 4.4	**<0.0001**	28.3 ± 3.0	32.2 ± 4.4	**<0.0001**
***STJ Ø, max***	29.8 ± 3.2	35.1 ± 5.1	**<0.0001**	30.3 ± 3.2	35.1 ± 5.1	**<0.0001**
***STJ Ø, average***	28.9 ± 3.0	33.7 ± 4.7	**<0.0001**	29.3 ± 3.1	33.7 ± 4.7	**<0.0001**
***STJ perimeter***	90.3 ± 11.3	104.9 ± 14.9	**<0.0001**	92 ± 9.7	104.9 ± 15.0	**<0.0001**
***STJ perimeter derived Ø***	28.9 ± 3.1	33.4 ± 4.7	**<0.0001**	29.3 ± 3.1	33.4 ± 4.8	**<0.0001**
***STJ area***	659.4 ± 140.2	887.1 ± 255.1	**<0.0001**	677.8 ± 143.8	887.1 ± 255.2	**<0.0001**
***STJ area derived Ø***	28.8 ± 3.0	33.3 ± 4.7	**<0.0001**	29.2 ± 3.1	33.3 ± 4.7	**<0.0001**
***LVOT Ø, min***	22.2 ± 2.6	23.5 ± 3.1	**0.009**	22.5 ± 2.5	23.5 ± 3.1	0.092
***LVOT Ø, max***	28.9 ± 2.8	29.9 ± 3.9	0.067	29.5 ± 2.8	29.9 ± 3.9	0.504
***LVOT Ø, average***	25.4 ± 2.9	26.7 ± 3.4	**0.014**	26.0 ± 2.5	26.7 ± 3.4	0.234
***LVOT eccentricity***	0.23 ± 0.06	0.22 ± 0.06	0.085	0.24 ± 0.05	0.22 ± 0.06	**0.043**
***LVOT perimeter***	80.7 ± 7.7	84.9 ± 11.4	**0.0014**	82.6 ± 8.1	84.9 ± 11.4	0.225
***LVOT perimeter derived Ø***	26.8 ± 16.8	27.0 ± 3.6	0.928	26.3 ± 2.6	27.0 ± 3.6	0.216
***LVOT area***	501.1 ± 97.9	560.9 ± 145.3	**0.006**	523.2 ± 102.1	560.9 ± 145.3	0.129
***LVOT area derived Ø***	25.1 ± 2.5	26.5 ± 3.5	**0.011**	25.7 ± 2.5	26.5 ± 3.5	0.187
***Impl. Angulation***	47.4 ± 8.1	53 ± 10.9	**0.001**	47.2 ± 7.8	53 ± 10.9	**0.003**
***Horizontal aorta (n/%)***	85/39.2	28/53.8	0.054	20/38.5	28/53.8	0.116
***Aortopathy***	16 (7.4%)	32 (61.5%)	**<0.0001**	3 (5.8%)	32 (61.5%)	**<0.0001**

**Table 9:** Detailed data of postprocedural outcomes (<72 h after the index procedure) of the study population and comparison between patients with tricuspid and bicuspid anatomy regarding unmatched and after propensity score matching. Outcomes based on VARC-2 definition. NA: not added value.

Postprocedural Outcomes (<72 h after the Index Procedure) of the Study Population (*n* = 269)		Unmatched (n = 269)			Matched (n = 52)		
		Tricuspid (n = 217)	Bicuspid (n = 52)	*p* Value	Tricuspid (n = 52)	Bicuspid (n = 52)	*p* Value
*Outcome*	*No. (%) of Events*						
***In-hospital mortality***	3 (1.1%)	3 (1.4%)	0	0.394	0	0	NA
***Device success***	267 (99.3%)	215 (99.1%)	52 (100%)	0.487	52 (100%)	52 (100%)	NA
***Myocardial infarction***	0	0	0	NA	0	0	NA
***Coronary obstruction***	0	0	0	NA	0	0	NA
***TIA***	1 (0.4%)	0	1 (1.9%)	0.041	0	1 (1.9%)	0.315
***Stroke***	3 (1.2%)	2 (0.9%)	1 (1.9%)	0.537	0	1 (1.9%)	0.315
***Acute kidney injure, stage 2 or 3***	5 (1.8%)	5 (2.3%)	0	0.269	1 (1.9%)	0	0.315
***Minor vascular complications***	19 (7.1%)	15 (6.9%)	4 (7.4%)	0.844	3 (5.7%)	4 (7.7%)	0.696
***Major vascular complications***	7 (2.6%)	5 (2.3%)	2 (3.8%)	0.530	0	2 (3.8%)	0.475
***Permanent Pacemaker Implantation***	76 (31.1%)	59 (30.4%)	17 (34%)	0.429	12 (24%)	17 (34%)	0.274
***Cardiac tamponade***	1 (0.4%)	1 (0.5%)	0	0.624	0	0	NA
***Annulus rupture***	0	0	0	NA	0	0	NA
***Valve malpositioning***	1 (0.4%)	1 (0.5%)	0	0.624	0	0	NA
***Need for a second valve***	0	0	0	NA	0	0	NA
***Postprocedural AR grade III or IV***	0	0	0	NA	0	0	NA

**Table 10:** Detailed data of outcomes at 30-day follow-up of the study population and comparison between patients with tricuspid and bicuspid anatomy regarding unmatched and after propensity score matching. Outcomes based on VARC-2 definition. NA: not added value.

VARC-2 Outcomes at 30 Days Follow-Up		Unmatched (n = 269)			Matched (n = 52)		
		Tricuspid (n = 217)	Bicuspid (n = 52)	*p* Value	Tricuspid (n = 52)	Bicuspid (n = 52)	*p* Value
*30-Day Cumulative Clinical Outcomes* *(n = 269)*	*No. (%) of Events*						
***All-cause mortality***	5 (1.8%)	5 (2.3%)	0	0.269	0	0	NA
***Cardiac mortality***	1 (0.4%)	1 (0.5%)	0	0.623	0	0	NA
***All stroke***	3 (1.2%)	2 (0.9%)	1 (1.9%)	0.537	0	1 (1.9%)	0.315
***Life-threatening bleeding***	1 (0.4%)	1 (0.5%)	0	0.623	0	0	NA
***Acute kidney injury, stage 2 or 3***	2 (2.4%)	2 (0.9%)	0	0.486	0	0	NA
***Coronary artery obstruction***	0	0	0	NA	0	0	NA
***Minor vascular complications***	19 (7.1%)	15 (6.9%)	4 (7.7%)	0.844	3 (5.7%)	4 (7.7%)	0.696
***Major vascular complications***	7 (2.6%)	5 (2.3%)	2 (3.8%)	0.530	0	2 (3.8%)	0.475
***New pacemaker implantation***	76 (31.1%)	59 (30.4%)	17 (34%)	0.429	12 (24%)	17 (34%)	0.274
***Valve-related dysfunction requiring repeat procedure (BAV, TAVI, or SAVR)***	0	0	0	NA	0	0	NA
***NYHA class III or IV***	2 (2.4%)	2 (0.9%)	0	0.621	0	0	NA
***Prosthetic valve endocarditis***	0	0	0	NA	0	0	NA
***Prosthetic valve thrombosis***	0	0	0	NA	0	0	NA
***Thrombo-embolic events***	0	0	0	NA	0	0	NA

**Table 11:** Echocardiographic parameters of the patients undergoing TAVI with tricuspid and bicuspid anatomy regarding unmatched comparison and after propensity score matching. NA: not added value. *: statistically significant difference.

	*Transthoracic Echocardiography Follow Up Data*								
	**Unmatched (n = 269)**			**Unmatched (n = 266)**			**Unmatched (n = 264)**		
	***Tricuspid Baseline*** ***(n = 217)***	***Bicuspid Baseline*** ***(n = 52)***	***p Value***	***Tricuspid Discharge (n = 214)***	***Bicuspid Discharge*** ***(n = 52)***	***p Value***	***Tricuspid 30-Days*** ***(n = 212)***	***Bicuspid 30-Days*** ***(n = 52)***	***p Value***
*Peak aortic gradient,* mmHg *	80.3 ± 25.7	79.9 ± 24.1	0.919	20.4 ± 7.7	20.3 ± 6.2	0.919	20.9 ± 7.8	19.9 ± 6.3	0.389
*Mean aortic gradient,* mmHg *	47.8 ± 15.5	47.6 ± 15.9	0.952	10.5 ± 4.3	10.1 ± 3.4	0.552	10.1 ± 4.3	9.7 ± 3.5	0.513
***LVEF,* %**	55.5 ± 12.7	54.2 ± 13.6	0.517	56 ± 9.7	56.4 ± 12.1	0.812	59.7 ± 10.3	59.1 ± 11.7	0.688
***Aortic regurgitation grade 2 or above***	81 (37.3%)	19 (36.5%)	0.916	12 (5.6%)	1 (1.9%)	0.269	4 (1.8%)	1 (1.9%)	0.986
***Paravalvular leakage trace/mild***	NA	NA	NA	4 (1.8%)	2 (3.8%)	0.389	8 (3.7%)	3 (5.7%)	0.519
***Paravalvular leakage moderate or above***	NA	NA	NA	2 (0.9%)	0	0.484	1 (1.9%)	0	0.620
	**Matched (n = 52)**			**Matched (n = 52)**			**Matched (n = 52)**		
	**Tricuspid Baseline** **(n = 52)**	**Bicuspid Baseline (n = 52)**	***p* Value**	**Tricuspid Discharge (n = 52)**	**Bicuspid Discharge** **(n = 52)**	***p* Value**	**Tricuspid 30-Days** **(n = 52)**	***Bicuspid 30-Days*** ***(n = 52)***	***p* Value**
*Peak aortic gradient,* mmHg *	79.8 ± 29.1	79.9 ± 24.1	0.985	21.3 ± 7.6	20.3 ± 6.2	0.451	21.1 ± 6.6	19.9 ± 6.3	0.333
*Mean aortic gradient,* mmHg *	47.5 ± 17.6	47.6 ± 15.9	0.976	10.9 ± 4.3	10.1 ± 3.4	0.285	9.9 ± 3.2	9.7 ± 3.5	0.339
***LVEF,* %**	55 ± 13.9	54.2 ± 13.6	0.771	55 ± 10.3	56.4 ± 12.1	0.531	60.3 ± 11.5	59.1 ± 11.7	0.601
***Aortic regurgitation grade 2 or above***	23 (44.2%)	19 (36.5%)	0.424	3 (5.7%)	1	0.308	1 (1.9%)	1 (1.9%)	1
***Paravalvular leakage trace/mild***	NA	NA	NA	3 (5.7%)	2	0.647	5 (9.6%)	3 (5.7%)	0.462
***Paravalvular leakage moderate or above***	NA	NA	NA	1 (1.9%)	0	0.315	1 (1.9%)	0	0.315

## Data Availability

The data presented in this study are available on request from the corresponding author. The data are not publicly available due to Hungarian legal regulations.

## Supplementary Materials

The following supporting information can be downloaded at https://www.mdpi.com/article/10.3390/jcm13020513/s1, Table S1, Data of the implantation depths regarding patients with and without PM implantation regarding the total patients cohort underwent TAVR procedure with Myval THV system.
