# Supplementary material for: Single-Center Experience with the Balloon-Expandable Myval Transcatheter Aortic Valve System in Patients with Bicuspid Anatomy: Procedural and 30-Day Follow-Up
Source: J Clin Med. 2024 Jan 17;13(2):513. doi: 10.3390/jcm13020513 (PMC10816957; doi:10.3390/jcm13020513)
Supplement: Supplementary file 1 [file jcm-13-00513-s001.zip › jcm-2792258-supplementary.pdf]

Supplementary Table

Implantation depth	PM (n=193)	non-PM (n=76)	p value
0-2 mm	2 (2.7%)	0 (0.0%)	0.023
2-4 mm	5 (6.7%)	28 (14.7%)	0.075
4-6 mm	33 (44.0%)	87 (45.5%)	0.819
6-8 mm	25 (33.3%)	57 (29.8%)	0.579
8-10 mm	10 (13.3%)	10 (5.2%)	0.024
>10 mm	0 (0.0%)	9 (4.7%)	0.056
4-6 or 6-8 mm	58 (77.3%)	144 (75.4%)	0.739

Supplementary Table S1, Data of the implantation depths regarding patients with and without PM implantation regarding the total patients cohort underwent TAVR procedure with Myval THV system.
